# Posttranslational Modifications of Ribosomal Proteins in Escherichia coli 

**Published:** 2011

**Authors:** M.V. Nesterchuk, P.V. Sergiev, O.A. Dontsova

**Affiliations:** Belozersky Institute of Physico-Chemical Biology, Lomonosov Moscow State University; Faculty of Chemistry, Lomonosov Moscow State University

**Keywords:** ribosomal proteins, posttranslational modification, *Escherichia coli *

## Abstract

А number of ribosomal proteins in*Escherichia coli*undergo posttranslational modifications. Six ribosomal proteins are methylated (S11, L3, L11, L7/L12, L16, and L33), three proteins are acetylated (S5, S18, and L7), and protein S12 is methylthiolated. Extra amino acid residues are added to protein S6. С-terminal amino acid residues are partially removed from protein L31. The functional significance of these modifications has remained unclear. These modifications are not vital to the cells, and it is likely that they have regulatory functions. This paper reviews all the known posttranslational modifications of ribosomal proteins in*Escherichia coli*. Certain enzymes responsible for the modifications and mechanisms of enzymatic reactions are also discussed.

## INTRODUCTION 

Ribosome is a sophisticated molecular machine that is responsible for the correct translation of genetic information from the mRNA nucleotide sequence into the amino acid sequence of the synthesized protein. A ribosome is a complex of ribosomal RNAs (rRNAs) and proteins consisting of two unequal parts (the large and the small subunits). The small subunit of *Escherichia coli* ribosomes consists of 16S rRNAs and 22 proteins (denoted as S1–S22); the large subunit consists of 5S and 23S rRNAs and 34 different proteins (L1–L36). Both rRNAs and ribosomal proteins undergo enzymatic modification in the cell. The N-terminal methionine residue is cleaved in more than half of ribosomal proteins. Six proteins are methylated (S11, L3, L11, L7/L12, L16, and L33), three proteins are acetylated (S5, S18, and L7), the S12 protein is methylthiolated, additional amino acid residues are added to the S6 protein, and the L31 protein undergoes partial proteolysis (L31) ( *Table 1* ). The nature of certain modifications of the ribosomal proteins of *E. coli* has yet to be determined [1].

Most genes encoding the enzymes that perform posttranslational modification have been identified. Nonetheless, the functional role of these modifications remains poorly studied. The central role of the ribosome in the mechanism of realization of the genetic information in the cell permits to assume that modified ribosomal proteins can be significant in a number of mechanisms of the regulation of gene expression. 

## PROCESSING OF THE N-TERMINUS OF RIBOSOMAL PROTEINS 

Removal of the N-terminal methionine residue by methionine aminopeptidase (MAP) is the most common type of posttranslational modifications in proteins. The terminal methionine is cleaved in 37 *E. coli* ribosomal proteins out of 57 ( *Table 2* ).

This modification is most frequently found in proteins in which the amino acid following methionine has the short side chain [2]. Large side chains impede the penetration of a protein into the active site of methionine aminopeptidase. If the second residue in a protein molecule is alanine (21 case) or leucine, proline, or glycine, the N-terminal residue is always cleaved. If the first residue is followed by lysine, isoleucine, glutamine, arginine, aspartic acid, tyrosine, glutamic acid, phenylalanine, or valine, as it is observed in 20 ribosomal proteins, the N-terminal methionine residue is always retained in the protein molecule. When position 2 is occupied by serine, in four cases out of five, the methionine residue is retained. It should be noted that methionine residues are cleaved only from a certain portion of protein L33 molecules; some chains (no more than 25%) remain with the N-terminal methylated methionine. It is possible that this is associated with the competition between N-terminal methylation and methionine cleaving [3].

## PROCESSING OF THE C-TERMINUS OF PROTEIN L31 

**Table 1:** Modifications of *E. coli* ribosomal proteins

Protein	Modification position	Modification	Modifying enzyme
S5	N-terminal amino group	Acetylation	RimJ
S6	C-terminus	Insertion of additional glutaminic acid residues	RimK
S11	N-terminal amino group	Methylation, formation of the isopeptide bond	Not determined
	Formation of isoaspartate residue	Not determined
S12	Asp88	Methylthiolation	RimO
S18	N-terminal amino group	Acetylation	RimI
L3	Gln150	Methylation	PrmB
L7/L12	Lys81	Methylation	Not determined
L11	Ala1, Lys3, Lys39	Methylation	PrmA
L12	N-terminal amino group	Acetylation	RimL
L16	N-terminal amino group	Methylation	Not determined
L31	C-terminus	Removal of amino acid residues	Not determined
L33	N-terminal amino group	Methylation	Not determined

The C-terminus amino acid sequence of the ribosomal protein L31 (...RFNK) determined chemically in [4] differs from the one predicted on the basis of the nucleotide sequence of the gene of this protein (…RFNKRFNIPGSK). A conclusion was made that protein L31 undergoes C-terminal processing (there may be a specific protease removing the RFNIPGSK fragment). These data were subsequently refuted; the primary structure of L31 was shown to agree with the genomic structure [5]. However, a mass spectrometry analysis of ribosomal proteins detects two peaks for L31: at 7871.1 Da, corresponding to the complete sequence of L31 predicted on the basis of the genomic sequence and that at 6971.1 Da, corresponding to the L31 fragment without the C-terminus region ...RFNIPGSK [1]. Protein L31 molecules are apparently only partially processed.

L31 is a component of a bacterial ribosome that has not been studied adequately. L31 is known to form a ribonucleoproteid complex with the rRNA proteins L5, L18, L25, and 5S. It is located at the vertex of the central protuberance in immediate proximity to the site of the subunit contact. It is probable that the posttranslational modification serves the purpose of protein activation or has a regulatory role. However, there is no data on the function of the site-specific proteolysis of L31 or on its possible mechanism. 

It is of interest that the *E. coli* genome contains two genes of protein L31 with a similar, but not identical, sequence [6]. Protein L31 that is present in cells under “regular” laboratory conditions in which culturing is performed contains the “zinc ribbon” motif. When there is a deficiency in zinc ions, the expression of another variant of L31 without the “zinc ribbon” is activated by the transcriptional regulator Zur. This switching likely facilitates economy of zinc ions in the cell. A similar mechanism has been described for ribosomal protein L36 [7].

## METHYLATION OF RIBOSOMAL PROTEINS 

Methylation is one of the most common types of posttranslational protein modifications to which various prokaryotic and eukaryotic proteins are subjected. Methylation is performed by special enzymes (methyltransferases), which use S-adenosylmethionine as a methyl group donor. Five classes of methyltransferases differing in structure and substrate specificity exist. 

The methylation of ribosomal proteins usually occurs at the side amino group of lysine or arginine; methylation of N-terminal amino groups is also common. Six ribosomal proteins are methylated in *E. coli* cells ( *Table 1* ) [8]. Methyltransferases that are specific to two proteins (L11 and L3) (PrmA and PrmB, respectively) have been identified; the genes encoding them have been found. Very little data exist about other modifications.

Certain methylated ribosomal proteins play a significant role in the functioning of ribosome: L7/L12 and L11 interact with the translation factors, and L3 participates in ribosome assembly. However, regardless of the fact that the functions of these ribosomal proteins have been studied appreciably well, the biological significance of their methylation is yet to be satisfactorily elucidated. Mutations in the genes encoding the corresponding methyltransferases do not result in noticeable phenotypical changes. The methylation apparently regulates the intra- and intermolecular interactions in a protein or impacts its affinity to RNA, thus influencing various cell processes: translation regulation, its accuracy, RNA processing, and ribosome assembly. 

**Protein L11 Methylation**

**Table 2:** Posttranslational removal of the N-terminal methionine residue in *E. coli* ribosomal proteins [1]

Protein	Removal of Met	The second residue following Met
S1	?	Thr
S2	+	Ala
S3	+	Gly
S4	+	Ala
S5	+	Ala
S6	–	Arg
S7	+	Pro
S8	+	Ser
S9	+	Ala
S10	–	Gln
S11	+	Ala
S12	+	Ala
S13	+	Ala
S14	+	Ala
S15	+	Ser
S16	–	Val
S17	+	Thr
S18	+	Ala
S19	+	Pro
S20	+	Ala
S21	+	Pro
S22	–	Lys
L1	+	Ala
L2	+	Ala
L3	–	Ile
L4	–	Glu
L5	+	Ala
L6	+	Ser
L7	+	Ser
L9	–	Gln
L10	+	Ala
L11	+	Ala
L12	+	Ser
L13	–	Lys
L14	–	Ile
L15	–	Arg
L16	–	Leu
L17	–	Arg
L18	–	Asp
L19	–	Ser
L20	+	Ala
L21	–	Tyr
L22	–	Glu
L23	–	Ile
L24	+	Ala
L25	–	Phe
L26	+	Ala
L27	+	Ala
L28	+	Ser
L29	–	Lys
L30	+	Ala
L31	–	Lys
L32	+	Ala
L33	+	Ala
L34	–	Lys
L35	+	Pro
L36	–	Lys

Ribosomal protein L11 is the most strongly methylated protein of the bacterial translation apparatus [9]. It contains three methylated amino acid residues: the N-terminal alanine residue is trimethylated at the α-amino group, the 3rd and 39th lysine residues are trimethylated at ε-amino groups. Thus, a total of nine methyl groups are posttranslationally bound to the protein [10]. The methylation is performed by a single enzyme, PrmA (protein modification), which has been isolated and characterized [11]. It was ascertained that this protein has a mass of 31 kDa and uses S-adenosylmethionine as a methyl group donor and preferentially modifies the unbound protein L11. The latter fact attests to the fact that methylation precedes the insertion of a protein into the ribosome [11].

The mutant *E. coli* strain containing no methyl groups in protein L11 was obtained. The position of the prmA gene encoding methyltransferase PrmA was determined using this strain [12].

This enzyme has a unique substrate specificity, which enables the modification of several amino groups of the protein which belong to different amino acid residues and are located at different sites with respect to the peptide backbone (α- and ε-amino groups). To do so, either the enzyme has to be bound to a substrate in several different orientations, or the system of flexible substrate positioning has to be used for the multiple modifications. This system facilitates the reorientation of the substrate with respect to the permanent binding site. The PrmA structure and the mechanism of its interaction with the substrate has been the subject of thorough study [13, 14].

**Fig. 1:**
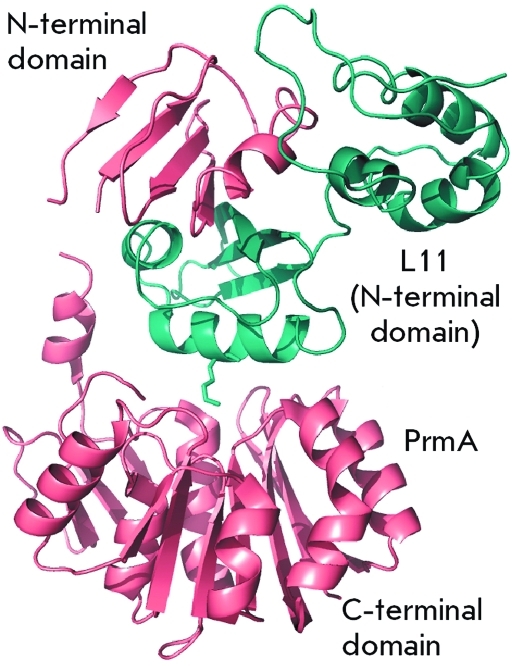
The PrmA–L11 complex structure. PrmA and L11 are colored in salmon and cyan. The Lys39 side chain is shown in a stick representation [13].

Methyltransferase PrmA consists of two domains connected by a flexible linker ( *Fig. 1* ). A large catalytic C-terminal domain is a typical example of class I methyltransferases. Seven-stranded β-sheet is flanked on both sides by α-helices. A small additional three-stranded β-sheet acts as an interlink between the C-terminal domain and the linker interdomain α-helix. The small N-terminal domain consists of a four-stranded β-sheet flanked by an interdomain linker α-helix, on one side, and by an N-terminal α-helix, on the other side. The N-terminal domain PrmA is unique; it is capable of recognizing and binding protein L11 ( *Fig. 1* ) [13]. It was ascertained using bioinformatics methods that the structure of the N-terminal domain PrmA is reminiscent of the V-domain of the EF-G factor, which is located in close proximity to protein L11 upon binding with the ribosome [15].

**Fig. 2:**
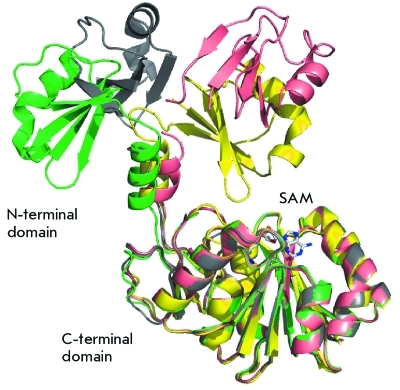
Superposition of four different conformations of PrmA. Apo-enzyme structures are colored in green and grey. The SAM-bound form of PrmA is colored in yellow. The structure of PrmA in the complex with L11 is colored in salmon [13].

The binding surface between the N-terminal domain PrmA and protein L11 is partially overlapped by the binding surface of L11 and 23S rRNA. Therefore, PrmA modifies L11 only in the unbound state, prior to its insertion into the ribosome. This fact is confirmed by the data obtained earlier on *in vitro* methylation [11]. The binding of the N-terminal domain PrmA to L11 is highly specific and is stabilized by a number of hydrogen bonds, whereas the catalytic C-terminal domain does not possess any specificity; its interaction with the substrate is stabilized only by the local hydrophobic interaction (the side chain of the modified lysine residue enters the active site via the tunnel formed by hydrophobic amino acid residues, which interact with the hydrophobic part of the side lysine radical). Due to the flexibility of the interdomain linker, the catalytic domain can change its position with respect to the N-terminal domain bound to L11 and methylate all the amino groups that are available to it ( *Fig. 2* ).

In the structure of a catalytic domain, there is a special hydrophobic pocket for the binding of S-adenosylmethionine; this pocket is open. This means that the exchange between S-adenosylhomocysteine (the reaction product) and S-adenosylmethionine is possible without disturbance of the enzyme-substrate complex. The active site of PrmA contains no atoms that could impede the rotation of the methylated amino group, which allows the enzyme that are bound to the substrate to trimethylate it. To methylate the amino group, it is necessary that the catalytic centre contain a basic amino acid residue that can accept a proton from the nitrogen atom. His104 located in the active site opposite the cofactor binding site apparently acts as such a residue. 

Thus, the methylation performed by enzyme PrmA is a rare example of simultaneous specific recognition of the target and multiple modifications of substrate due to the spatial separation of the binding site and the catalytic center, as well as their mutual mobility. A single methyltransferase molecule is capable of sequentially introducing nine methyl groups into protein L11, without the dissociation of the enzyme-substrate complex. 

Protein L11 is a conservative component of the large subunit of the bacterial ribosome and is an active participant of the interaction between the ribosome and the factors of translation initiation, elongation, and termination. It consists of two domains connected by a flexible linker: the C-terminal domain binding 23S rRNA and the N-terminal domain interacting with the translation factors [13]. The N-terminal domain is the target of antibiotic thiostrepton, which inhibits EF-G–ribosome binding (resistance towards thiostrepton is ensured by a number of mutations in the N-terminal domain of protein L11) [16]. It was revealed via cryo-electron microscopy that the N-terminal domain of L11 is in direct contact with the EF-G [15] and EF-Tu factors [17].

All amino acid residues trimethylated with PrmA are located in the N-terminal domain. This arrangement of the modified residues near the site of contact with the translation factors may point to the functional significance of methylation. The PrmA structure is conservative in all bacteria, which may also attest to its contribution to L11 functions. Nevertheless, the function of modification carried out by PrmA has not, as yet, been ascertained. In addition to being viable, the strain with a mutation of the prmA gene does not differ noticeably from the wild-type strain (the same growth rate and the same behavior under stress conditions) [18, 19]. This means that multiple methylation of L11 is not necessary for the normal functioning of the ribosome. Meanwhile, it may have an effect both on the rate and accuracy of such stages of ribosome functioning as decoding and translocation, which can be detected only using a very accurate kinetic *in vitro* analysis or by the *in vivo* introduction of specific mutations into protein L11 or other components of the translation apparatus [13].

**Methylation of Protein L3**

**Fig. 3:**
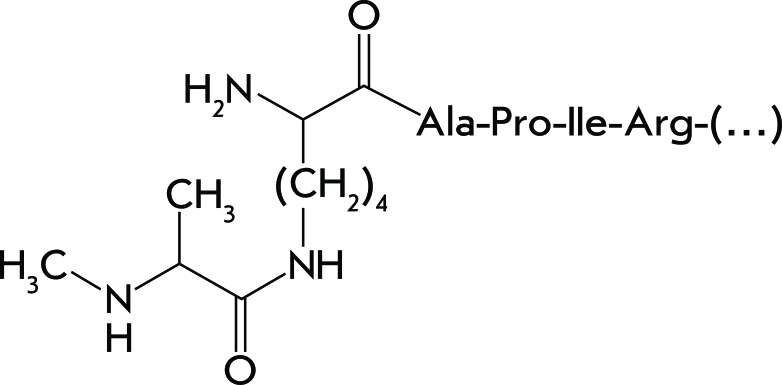
Structure of the N-terminal amino acid residue of S11, which is methylated and forms isopeptide linkage [25].

One methyl group is posttranslationally added to ribosomal protein L3 [20]. The methylation takes place at the amide group of the 150th glutamine residue [21]. Modification is performed by specific methyltransferase PrmB. Its gene has been identified [12]. PrmB is the first described methyltransferase with an amide nitrogen atom acting as its target.

The absence of methylation in protein L3 in the strain containing a mutation in the prmB gene is combined with cold sensitivity. The growth rate of mutant cells at 22°С is considerably lower than that in wild-type cells [22, 23]. This is due to the fact that at low temperatures ribosome assembly in mutant cells is inefficient; the structure and stability of the resulting intermediate ribonucleoproteid complexes differ from that of the corresponding intermediates in the wild-type strain. Nevertheless, the mutant ribosomes that are completely formed at a reduced temperature do not differ from the wild-type ribosomes in terms of their stability. No difference in the translation rate or its accuracy was observed neither *in vivo* nor *in vitro* [22].

*In vitro* studies of the activity of the PrmB enzyme demonstrated that protein L3, in the unbound state, does not undergo methylation. Neither does methyltransferase methylate, the completely assembled ribosome. The highest activity is observed in the partially assembled ribosome and in the presence of RNA (of any type, not necessarily ribosomal) in the reaction mixture [22].

Protein L3 is bound to the 3’-terminal site of 23S rRNA at the very first stage of structure folding and, along with L24, is the initiator of the entire process of ribosome assembly [24].

A conclusion can be made from the aforementioned that PrmB *in vivo* methylates L3 by binding to the ribosome at one of the intermediate stages of its folding; hence, it is likely to have a certain effect on the correctness of its packing. Thus, the PrmB enzyme can be referred to ribosome assembly factors.

Protein L3 has a globular domain located on the ribosome surface and a long tail that is deeply submerged inside. The modified 150th glutamine residue is located inside the ribosome near the tunnel or the growing polypeptide chain. It forms contacts with the nucleotide residues G2032, C2055, and A2572, which are located in the 3’-terminal region of 23S rRNA. This residue apparently contributes to the formation and maintenance of the correct rRNA conformation. 

**Methylation of Protein S11 and Formation of the Isomeric Peptide Bond**

In ribosomal protein S11, the N-terminal amino group belonging to the alanine residue undergoes methylation. In addition to methylation, the formation of an isopeptide bond is observed. Only one *E. coli * protein, S11, is susceptible to this transformation. During this process, the peptide bond between the first and second residues (alanine and lysine) in the S11 molecule is destroyed ( *Fig. 3* ) [25].

It was reported [26] that the isoaspartate residue was detected in the protein S11; there is 0.5 mol of isoaspartate per 1 mol of the protein. It was demonstrated that only S11 has such a modification in the logarithmic growth phase.

Neither the enzymes that perform the aforementioned modifications nor the functional role of the modifications have been ascertained. 

Protein S11 is located in immediate proximity to mRNA and tRNA in the E-site. Its N-terminus protruding on the ribosome surface cannot be seen in the crystal structure and probably has no fixed orientation. It is unlikely that the modified residue contributes to the maintenance of the ribosome structure. The methylated N-terminus of S11 probably interacts with tRNA and facilitates its escape from the E-site. 

**Methylation of Protein L7/L12**

Ribosomal protein L7/L12 is monomethylated at the ε-amino group of the 81st lysine residue. The degree of methylation strongly depends on temperature. At 37°С, almost no modification is observed (less than 0.1 methyl groups per protein molecule). The number of groups introduced increases abruptly with decreasing temperature and is equal to 0.6 monomethyl-lysine residues per protein molecule [27]. The enzyme performing this reaction has not been identified.

The protein L7/L12 is located in the ribosome as a tetramer representing a rod-shaped appendix, the so-called “L7/L12 stalk”. Each chain in a tetramer consists of two domains: the N-terminal domain, which is bound to protein L10, and the C-terminal domain. The domains are connected by a flexible linker, which makes it possible for the C-terminal domains to change their orientation with respect to the large subunit. Thus, L7/L12 is the only ribosomal protein that does not have direct contact with rRNA; it is bound to it via the complex with protein L10. This complex plays a significant role in the translation process; it participates in the binding between the translation factors (IF2, EF-Tu, EF-G, and RF3) and the ribosome [28]. The methylated residues are located in the C-terminal domain, and it is possible that they contribute to the interaction with the translation factors.

**Methylation of Proteins L16 and L33**

N-terminal amino groups are methylated in the ribosomal proteins L16 and L33. In L16, the first methionine residue is methylated [29]. In L33, some polypeptide chains start with the monomethylated methionine (no more than 25%), whereas some chains start with monomethylated alanine [30]. Such heterogeneity is probably associated with the competition between the processes of methylation and N-terminal methionine cleavage [30]. The assumption of a possible reduction of N-formylmethionine to N-methylmethionine has been refuted [3].

The methyltransferases performing the modification of proteins L16 and L33 have not been identified. 

Another modification type has been detected in protein L16. Based on the amino acid sequence, the molecular weight of protein L16 is supposed to be 15281.3 Da. However, there is no peak in the mass spectrum of this protein that would correspond to this weight, although, there is a peak corresponding to 15326.2 Da, which is higher by 44.9 Da. The methyl group at the N-terminus of the protein increases its weight only by 14 Da. This entails that the molecule L16 should contain at least one more posttranslational modification. Hypothetically, Arg81 undergoes hydroxylation. However, in this case the modified protein should be lighter than the value observed by mass spectroscopy: 14.9 Da [1]. Another methylation or hydroxylation may take place. The more explicit data concerning the nature and localization of the unknown modification were obtained from the mass spectra of the products of tryptic cleavage of protein L16.

Proteins L16 and L33 are located near the central protuberance on the opposite sides from it. Their N-terminal residues are exposed to the ribosome surface and are not in direct contact with rRNA and the proteins. 

## ACETYLATION OF RIBOSOMAL PROTEINS 

N ^α^ -acetylation of proteins is catalysed by N ^α^ -acetyltransferases, which transfer the acetyl group from acetylcoenzyme A to the N-terminal amino group of the protein. In eukaryotes, this modification of proteins is widespread: 80–90% of cytoplasmic proteins in mammals and 50% in yeasts are acetylated at the N-terminal amino acid residue [31]. In prokaryotes, this modification is rarely realized. Only four *E. coli* proteins are known to undergo this process: the EF-Tu factor and the ribosomal proteins S5, S18, and L7. The genes which encode the enzymes that perform the modification of the ribosomal proteins S5, S18, and L7 were determined: rimJ, rimI, and rimL, respectively. Each of the mentioned transferases specifically modifies only one protein (as opposed to eukaryotes, in which these enzymes are less specific). Despite their similar functions, the structures of these enzymes are very different. The similarity between sequences *RimI* (148 residues), *RimJ* (194 residues), and *RimL* (178 residues) is 19 and 20%, respectively; while that between RimJ and RimL is 23% (although RimI and RimJ are alanine acetyltransferases, whereas RimL is serine acetyltransferase). It is likely that these proteins have no common ancestor and evolved independently from each other [32].

Eukaryotic N ^α^ -acetyltransferases typically consist of two or three different subunits; they cotranslationally modify the substrate, whereas the prokaryotic enzymes are most frequently monomeric or in the form of homodimers (e.g., RimL) and acetylate substrate posttranslationally [33].

**Acetylation of Protein S5**

Ribosomal protein S5 is acetylated at the α-amino group of the first alanine residue [34]. The acetylation is performed by a specific enzyme, RimJ (ribosomal modification) [35]. The *rimJ* gene encoding this enzyme has been identified and sequenced [36].

The substrate specificity of RimJ has been ascertained. Poot *et al* . [37] investigated an *E. coli* strain containing the mutation in the central pseudoknot in 16S rRNA (C18A). This mutation leads to the disturbance of the assembly of the 30S subunit, deterioration of binding between it and proteins S1, S2, S18, and S21 and, therefore, to the reduction in translation efficacy. Moreover, the mutation results in a decrease in the proportion of acetylated molecules S5; i.e., the activity of RimJ decreases. This is apparently connected with the fact that S5 is located in immediate proximity to the central pseudoknot, in which the mutations can change the site of landing of S5 on the 30S subunit; therefore, RimJ cannot be bound to the substrate. It should be noted that non-acetylated S5 has not been found in the assembled 70S mutant ribosomes. The binding between the mutant 30S subunit and 50S apparently stabilizes the functionally active conformation of the 30S subunit. Having this conformation, the 30S subunit becomes the RimJ substrate. It was earlier demonstrated that the mutation in protein S4 also results in lower efficacy of acetylation of S5 [38]. These data attest to the fact that acetylation of S5 is performed on an assembled ribosome.

**Fig. 4:**
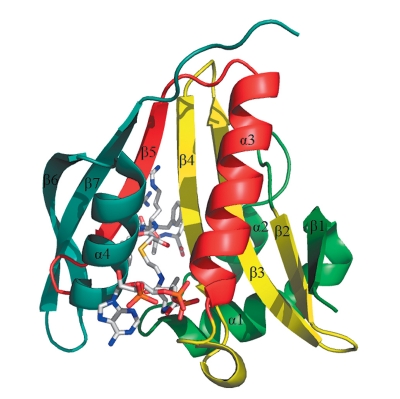
Structure of the RimI-bisubstrate complex. Bisubstrate is shown as sticks. From the N-terminus, secondary structural elements are colored green (b1, a1, a2), yellow (b2–b4), red (a3, b5), and blue (a4, b6) [32].

The function of RimJ is associated not only with the modification of protein S5, but also with other stages of biogenesis of the small ribosomal subunit [39]. In the *E. coli* strain with mutation in the gene of protein S5 (the 28th glycine residue is substituted for aspartic acid), the assembly of ribosomes is disturbed, translation accuracy is reduced, and cold sensitivity is observed. The superexpression of RimJ in this strain completely recovers all the translational defects. This entails that, regardless of the acetyltransferase actrivity, RimJ contributes to the formation of the correct ribosome structure. It can be proven by the fact that RimJ is bound to subunit 30S at early stages of its assembly [39]. The functions of RimJ as a factor of ribosome and acetyltransferase assembly can be combined and performed simultaneously or sequentially.

Protein S5 is located on the side of the small subunit that is opposite to the decoding centre. The N-terminal residues protrude over the ribosome surface and cannot be seen in the crystal structure. Therefore, the α-amino group of the first residue of protein S5 within a ribosome is accessible for acetylation, which agrees with the results of study [37].

Protein RimJ performs functions that are not directly connected with the acetylation of S5. It has been demonstrated that RimJ is a repressor of the pap operon responsible for pilus biosynthesis in the pathogenic *E. coli* strain causing pyelonephritis. RimJ regulates the transcription of this operon depending on environmental conditions (temperature, the presence of nutrients). The mechanism of this regulation and how it is connected to the acetyltransferase function of RimJ have not been ascertained [40].

**Acetylation of Protein S18**

In a similar fashion to S5 and L12, protein S18 undergoes N-terminal acetylation (at the alanine residue) [41]. The modification is performed by the specific acetyltransferase encoded by the *rimI* gene [36].

Acetylation of S18 does not belong to the modifications that are vital to a cell. The cells with mutations in the *rimI* gene are not only viable, but they also do not phenotypically differ from wild-type cells [42].

The 3D structure of RimI from *Salmonella typhimurium* (the primary structure being absolutely identical to RimI from *E. coli* ) has been determined [32]. The enzyme has a mixed αβ-structure, with the central seven-stranded β-sheet flanked by four α-helices ( *Fig. 4* ). The central sheet has a predominately antiparallel structure, with the exception of the parallel 4th and 5th strands. The order of β-strands in the sheet is linear, with the exception of strand β7 that is located between β5 and β6. The sheet has a V-shaped structure, where strands β1–β4 form one shoulder, while strands β5–β7 form the other shoulder. It is assumed that the acetyltransferase center is located in the V-shaped broadening between the β4- and β5-strands.

**Fig. 5:**
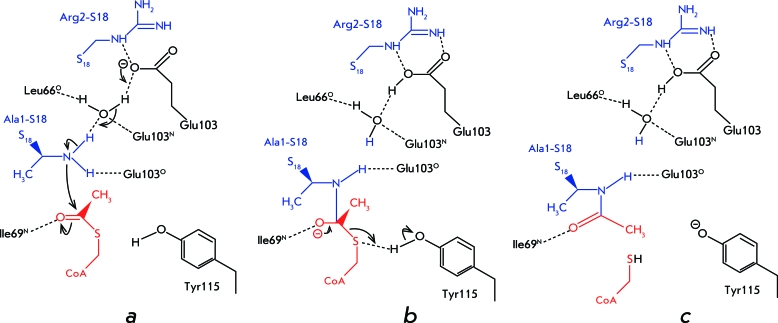
The proposed mechanism of the reaction catalyzed by RimI. (i) Nucleophilic attack on the carbonyl carbon of AcCoA; (ii) collapse of the tetrahedral intermediate; (iii) product complex [32].

Based on the data on the 3D structure of the complex of enzyme with substrate and coenzyme (acetyl coenzyme A), a mechanism of acetyltransferase reaction was proposed ( *Fig. 5* ).

The N-terminal nitrogen atom in S18 nucleophilically attacks the carbonyl carbon atom of acetyl coenzyme A, with the Glu103 residue acting as a proton acceptor ( *Fig. 5a* ). This results in the formation of a tetrahedral intermediate ( *Fig. 5b* ). Tyr115 acts as a proton donor upon decomposition of the intermediate ( *Fig. 5c* ).

Despite the fact that the mechanism of S18 acetylation has been ascertained, the function of acetylation and the stage at which it takes place have not as yet been elucidated. Protein S18 is located in the central domain of the small ribosomal subunit near proteins S11 and S6. The interaction between S18 and S6 is so strong that a stable heterodimer is formed [43]. S18 is located near the E-site. The first 15 N-terminal residues in S18 cannot be seen in the crystal structure of the ribosome; therefore, it is most likely that they do not have a fixed 3D position. They are probably located near the site of mRNA landing on the small subunit. In this case, the N-terminal acetylation can have an effect on the translation initiation process.

**Acetylation of Protein L12**

Ribosomal protein L12 exists in two different forms: the non-acetylated (L12) and acetylated (so-called L7) forms [44]. Due to the identity of the amino acid sequences, this protein is called L7/L12.

Acetylation of the α-N-atom of Ser1 in protein L12 is performed by the specific enzyme RimL and results in the formation of L7 [45]. RimL is capable of *in vitro* acetylation of the two unbound proteins L12 [46] and L12 within the ribosome [33]. Apparently, *in vivo* modification of L12 can also take place at any stage of ribosome biogenesis. As opposed to the totally modified S5 and S18, L12 is only partially acetylated. The L7/L12 ratio varies depending on the phase and rate of cell growth. In the middle of the logarithmic phase, the proportion of L12 attains 85%; then, the L7 content gradually increases to 75–80% in the stationary phase [47]. When the cells grow in a minimum medium, all of the protein is converted into the L7 form.

**Fig. 6:**
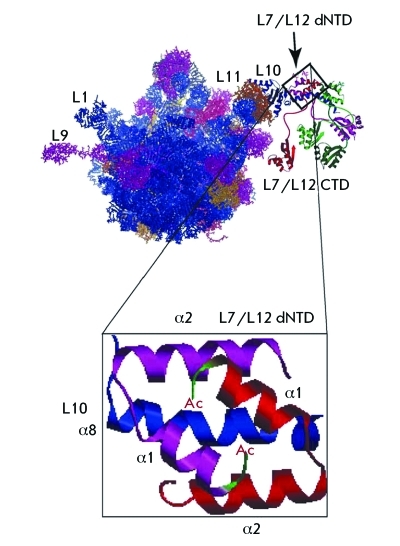
A hypothetical model of *E. coli* ribosomes with the ribosomal stalk complex attached. L10 is shown in blue; L7/L12 dimers, in red/magenta and light/dark green. The detailed view of the interaction of the L7/L12 NTD dimer with a segment of the α8 helix of L10 illustrates the hypothesis that N-terminal acetylation results in stronger binding of the L7 NTD dimer onto the α8 helix of L10 [48].

The modification of L12 was shown to enhance the strength of the complex formed by the L7/L12 tetramer and protein L10 [48]. These authors account that by the fact that acetylation stabilizes the N-terminal α-helices of L7 (are denoted as α1 in *Fig. 6* ) and fix the position of the N-terminal residue in space, thus making the structure more compact and stronger.

Nevertheless, the strain with a mutation in the *rimL* gene, in which the entire protein L12 is present in deacetylated form, has no noticeable phenotypic distinctions from the wild-type strain. In particular, there is no difference in the cell growth rate at 25, 37, or 42°С [46]. Therefore, the modification is insignificant for ribosome functioning; the question as to its possible function remains open.

The substrate specificity of RimL and nature of the N-terminal amino acid residue of the substrate was studied in [33]. According to the data published, in N ^α^ -acetylated proteins these residues typically are represented by serine, alanine, or methionine. Thus, it is serine for L12 from *E. coli* and alanine for L12 from *Pseudomonas aeruginosa* and *Bacillus subtilis* and S18 and S5 from *E. coli* . The assumption that RimL is not specific towards the N-terminal residue has been experimentally confirmed. RimL efficiently acetylates *in vitro* the mutant L12, with Ser1 replaced by Ala1 [33].

In the case of eukaryotic N ^α^ -acetyltransferases, the second amino acid residue of the N ^α^ -acetylated protein has an effect on the modification of the first protein. If the second residue is aspartate or glutamate, modification occurs efficiently. In order to investigate the effect of the second amino acid residue on the activity of RimL, the mutant L12 was obtained, with Ile2 replaced by Asp2. It turned out that RimL acetylates this mutant protein much less efficiently in comparison with the native L12. This, again, emphasizes the difference between prokaryotic N ^α^ -acetyltransferases and eukaryotic ones [33].

**Fig. 7:**
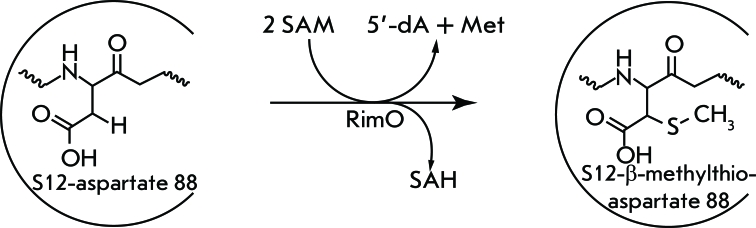
Reaction catalysed by RimO. The methylthiolation of aspartyl 88 of protein S12.

The crystal structure of RimL was obtained from *Salmonella typhimurium* (the similarity between the primary structure and the RimL from *E. coli* is 83%). RimL is a homodimer that is capable of binding two molecules of acetyl coenzyme A and modifying dimeric L12 [49].

## METHYLTHIOLATION OF PROTEIN S12 

The primary structure of ribosomal protein S12 of *E. coli* was determined chemically; however, its 88th amino acid residue could not be identified [50]. The subsequent sequencing of the gene encoding S12 demonstrated that aspartic acid is located at this position [51]. Only 20 years later was it shown mass spectrometrically that the molecular weight of S12 is equal to 13652 Da (which is bigger than that predicted on the basis of the nucleotide sequence by 46.1 Da) [1]. Further study of this phenomenon demonstrated that this discrepancy is conditioned by the presence of the methylthioether group (–SCH3) ( *Fig. 7* ) at the β-carbon atom of Asp88 [52]. Later, the *rimO* gene encoding methylthiotransferase that performs this posttranslational modification was found [53].

This reaction is an example of the enzymatic formation of the C–S bond from C–H, which is relatively rare in nature. These reactions occur via a radical mechanism using S-adenosylmethionine as a coenzyme [54].

S12 is the conservative element of ribosome; the Asp88 residue was found in all known S12 homologues: in bacteria, archaebacteria and eukaryotes (although the modification is not always observed). Asp88 is located near the functional centers of the ribosome. The attempts to obtaining *E. coli* cells with a mutation of this amino acid were unsuccessful. All these facts point to the significance of Asp88 in the functioning of ribosome.

All the methylthiotransferases that had been known before RimO posttranscriptionally modify RNA; RimO is the first studied enzyme in this family, with a protein as its target. In addition to RimO, only one methylthiotransferase was detected in *E. coli* : MiaB modifying tRNA. The amino acid sequences of these two proteins are characterized by a close similarity [53]. In particular, they both contain the СхххСххС motif, which is canonical for the entire methylthiotransferase family.

**Fig. 8:**
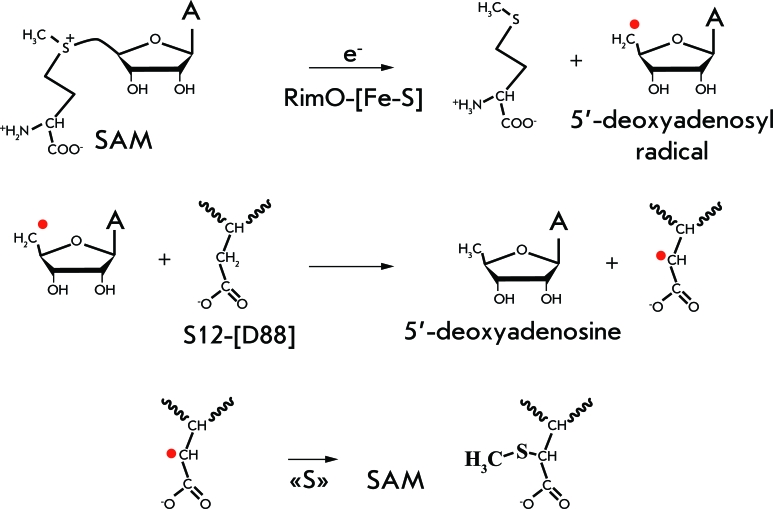
Mechanism of the reaction catalysed by RimO [55].

The methylthiolation of protein S12 was carried out via a radical mechanism ( *Fig. 8* ). At the first stage, the C–S bond in S-adenosylmethionine is broken, yielding an unbound methionine and a 5’-desoxyadenosyl radical. Then, this radical takes away a hydrogen atom from the β-carbon atom of Asp88. Next, a thioether is formed, which undergoes methylation at the final stage. Thus, two molecules of S-adenosylmethionine are required to modify a single molecule of protein S12 [55].

At the final stage, the RimO enzyme also takes part in the methylation. This means that RimO is methyltransferase, although no conservative S-adenosylmethionine-binding motifs that are typical of enzymes of this class have been detected in it [56].

**Fig. 9:**
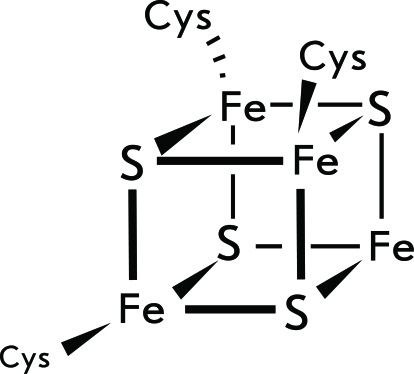
The [4Fe-4S] cluster coordinated by cysteine residues in the structure of RimO [56].

It was ascertained through spectroscopic studies that there are two [4Fe–4S] clusters in the structure of RimO ( *Fig. 9* ). The first cluster is coordinated by the residues Cys150, Cys154, and Cys157 (the conservative СхххСххС motif); the second is coordinated by the residues Cys17, Cys53, and Cys82. It is assumed that the first cluster participates in the formation of the 5’-desoxyadenosyl radical, whereas the second one serves as the source of sulphur atoms for thioether formation [56].

RimO contains the so-called TRAM domain, which serves for RNA binding in MiaB [53]. This could be an indication that RimO modifies protein S12 within the ribosome. It has indeed been confirmed experimentally that RimO *in vivo* methylthiolates residue Asp88 of protein S12, which is a component of the small subunit [57]. It was ascertained earlier that the recombinant RimO from *E. coli* and *Thermatoga maritima* can modify *in vitro* the synthetic peptide substrate imitating a loop containing residue Asp88; however, the efficacy of the modification is very low [58].

It was recently demonstrated that in addition to RimO, the conservative protein YcaO also participates in the modification of protein S12; the function of YcaO remained unknown. Knockout of the *ycaO* gene results in the almost complete suppression of the methylthiolating activity of RimO. Moreover, the transcriptome analysis of strains with the deletion of the *rimO* and *ycaO* genes points to the overlapping of transcriptional phenotypes, which attests to the functional similarity between RimO and YcaO. Protein YcaO is bound to the small subunit and probably functions as a chaperon by facilitating the formation of the enzyme–substrate complex [57].

It should be mentioned that after the methylthiolation of the aspartic acid residue, a new chiral centre (β-carbon atom) emerges; however, its configuration has not yet been ascertained. 

Protein S12 consists of a globular domain located near the A-site in the decoding centre and a long rod-shaped tail, which fixates the protein on the small subunit. S12 is the only protein located on the contact surface between the large and small subunits. The modified residue is located near the decoding centre; however, it forms direct contacts neither with mRNA nor tRNA. It is submerged into a pocket formed by two loops: the first loop is formed from the nucleotides 522–528 of 16S rRNA, the second loop is formed from the amino acid residues 44–51 of S12. 

The function of S12 modification has not been elucidated yet. It is known that mutations in the neighboring residues (Lys87, Ley89, Pro90, Gly91, and Arg93) lead to the emergence of resistance to streptomycin or streptomycin dependence [53]. It is also known that protein S12 participates in the spontaneous translocation of the ribosome (independent of EF-G and GTP), whereas the mutation in the neighboring 87th amino acid residue violates this function [59]. Nevertheless, in case of mutation in the *rimO* gene, none of these phenotypical changes take place. The only difference between this mutant and the wild-type strain consists in a slightly reduced growth rate [53].

## MODIFICATION OF PROTEIN S6 

Ribosomal protein S6 has a unique type of posttranslational modification. From two to six glutaminic acid residues (…ADDAEAGDSEE(E) _0–4_ ) are located on its C-terminus [60, 61]. Only the first two (…ADDAEAGDSEE) are encoded in the gene ( *rpsF* ) [62]; the rest of the residues are added posttranslationally. The modification occurs in a stepwise fashion; glutaminic acid residues being added one by one [63].

The mutant strain was obtained; it contained only two glutaminic acid residues and demonstrated no heterogeneity of the C-terminus of protein S6. Using this strain, the *rimK* gene that is responsible for this modification was detected [64]. This gene encodes an enzyme with a molecular weight of 31.5 kDa, which recognizes the protein S6 and adds more residues on its C-terminus. In the case of the mutation in the *rpsF* gene, resulting in substitution of the penultimate residue of glutaminic acid by lysine, no posttranslational modification of protein S6 was observed [64]. This means that RimK recognizes the C-terminal region of wild-type protein S6. In some mutant strains, RimK adds more than four glutaminic acid residues to S6; however, the reasons for this have not been elucidated [65].

It was ascertained that under conditions when ribosome assembly does not occur (in cells irradiated with ultraviolet light), S6 does not undergo modification [66]. The RNA-binding motif was found in RimK using bioinformatics methods [67]. These data may point to the fact that modification occurs during or after the insertion of protein S6 into the ribosome.

Protein S6 is located in the central domain of the small ribosomal subunit. By interacting with the proteins S18, S8, and S15, it protects 16S rRNA against attacks by endonucleases. The C-terminal amino acid residues of S6 protrude outside and are not seen in the crystal structure. 

S6 is the most acidic protein of the 30S subunit (p *I * = 4.8), the posttranslational modification enhancing its acidity to a larger extent. The function of this modification has yet to be elucidated; however, it is the first known case of posttranslational addition of amino acid residues.

## References

[1] Arnold R.J., Reilly J.P. (1999). Anal. Biochem..

[2] Ben-Bassat A., Bauer K., Chang S.Y., Myambo K., Boosman A., Chang S. (1987). J. Bacteriol..

[3] Chang F.N., Budzilowicz C. (1977). J. Bacteriol..

[4] Brosius J. (1977). Biochemistry..

[5] Eistetter A.J., Butler P.D., Traut R.R., Fanning T.G. (1999). FEMS Microbiol. Lett..

[6] Makarova K.S., Ponomarev V.A., Koonin E.V. (2001). Genome Biol..

[7] Gabriel S.E., Helmann J.D. (2009). J. Bacteriol..

[8] Polevoda B., Sherman F. (2007). Mol. Microbiol..

[9] Chang C.N., Chang N. (1975). Biochemistry..

[10] Dognin M.J., Wittmann-Liebold B. (1980). Eur. J. Biochem..

[11] Chang F.N., Cohen L.B., Navickas I.J., Chang C.N. (1975). Biochemistry..

[12] Colson C., Lhoest J., Urlings C. (1979). Mol. Gen. Genet..

[13] Demirci H., Gregory S.T., Dahlberg A.E., Jogl G. (2007). EMBO J..

[14] Demirci H., Gregory S.T., Dahlberg A.E., Jogl G. (2008). Structure..

[15] Agrawal R.K., Linde J., Sengupta J., Nierhaus K.H., Frank J. (2001). J. Mol. Biol..

[16] Cameron D.M., Thompson J., March P.E., Dahlberg A.E. (2002). J. Mol. Biol..

[17] Valle M., Zavialov A., Li W., Stagg S.M., Sengupta J., Nielsen R.C., Nissen P., Harvey S.C., Ehrenberg M., Frank J. (2003). Nat. Struct. Biol..

[18] Vanet A., Plumbridge J.A., Guerin M.F., Alix J.H. (1994). Mol. Microbiol..

[19] Colson C., Smith H.O. (1977). Mol. Gen. Genet..

[20] Lhoest J., Colson C. (1977). Mol. Gen. Genet..

[21] Muranova T.A., Muranov A.V., Markova L.F., Ovchinnikov Y.A. (1978). FEBS Lett..

[22] Lhoest J., Colson C. (1981). Eur. J. Biochem..

[23] Heurguе-Hamard V., Champ S., Engstrom A., Ehrenberg M., Buckingham R.H. (2002). EMBO J..

[24] Kaczanowska M., Ryden-Aulin M. (2007). Microbiol. Mol. Biol. Rev..

[25] Chen R., Chen-Schmeisser U. (1977). Proc. Natl. Acad. Sci. USA..

[26] David C.L., Keener J., Aswad D.W. (1999). J. Bacteriol..

[27] Chang F.N. (1978). J. Bacteriol..

[28] Gudkov A.T. (1997). FEBS Lett..

[29] Brosius J., Chen R. (1976). FEBS Lett..

[30] Chang C.N., Schwartz M., Chang F.N. (1976). Biochem. Biophys. Res. Commun..

[31] Polevoda B., Sherman F. (2002). Genome Biol..

[32] Vetting M.W., Bareich D.C., Yu M., Blanchard J.S. (2008). Protein. Sci..

[33] Miao L., Fang H., Li Y., Chen H. (2007). Biochem. Biophys. Res. Commun..

[34] Wittmann-Liebold B., Greuer B. (1978). FEBS Lett..

[35] Janda I., Kitakawa M., Isono K. (1985). Mol. Gen. Genet..

[36] Yoshikawa A., Isono S., Sheback A., Isono K. (1987). Mol. Gen. Genet..

[37] Poot R.A., Jeeninga R.E., Pleij C.W., van Duin J. (1997). FEBS Lett..

[38] Cumberlidge A.G., Isono K. (1979). J. Mol. Biol..

[39] Roy-Chaudhuri B., Kirthi N., Kelley T., Culver G.M. (2008). Mol. Microbiol..

[40] White-Ziegler C.A., Black A.M., Eliades S.H., Young S., Porter K. (2002). J. Bacteriol..

[41] Yaguchi M. (1975). FEBS Lett..

[42] Isono K., Isono S. (1980). Mol. Gen. Genet..

[43] Recht M.I., Williamson J.R. (2001). J. Mol. Biol..

[44] Terhorst C., Moller W., Laursen R., Wittmann-Liebold B. (1973). Eur. J. Biochem..

[45] Tanaka S., Matsushita Y., Yoshikawa A., Isono K. (1989). Mol. Gen. Genet..

[46] Isono S., Isono K. (1981). Mol. Gen. Genet..

[47] Ramagopal S., Subramanian A.R. (1974). Proc. Natl. Acad. Sci. USA..

[48] Gordiyenko Y., Deroo S., Zhou M., Videler H., Robinson C.V. (2008). J. Mol. Biol..

[49] Vetting M.W., de Carvalho L.P., Roderick S.L., Blanchard J.S. (2005). J. Biol. Chem..

[50] Funatsu G., Yaguchi M., Wittmann-Liebold B. (1977). FEBS Lett..

[51] Post L.E., Nomura M. (1980). J. Biol. Chem..

[52] Kowalak J.A., Walsh K.A. (1996). Protein. Sci..

[53] Anton B.P., Saleh L., Benner J.S., Raleigh E.A., Kasif S., Roberts R.J. (2008). Proc. Natl. Acad. Sci. USA..

[54] Booker S.J., Cicchillo R.M., Grove T.L. (2007). Curr. Opin. Chem. Biol..

[55] Fontecave M., Mulliez E., Atta M. (2008). Chem. Biol..

[56] Lee K.H., Saleh L., Anton B.P., Madinger C.L., Benner J.S., Iwig D.F., Roberts R.J., Krebs C., Booker S.J. (2009). Biochemistry..

[57] Strader M.B., Costantino N., Elkins C.A., Chen C.Y., Patel I., Makusky A.J., Choy J.S., Court D.L., Markey S.P., Kowalak J.A. (2011). Mol. Cell. Proteomics..

[58] Arragain S., Garcia-Serres R., Blondin G., Douki T., Clemancey M., Latour J.M., Forouhar F., Neely H., Montelione G.T., Hunt J.F. (2010). J. Biol. Chem..

[59] Asatryan L.S., Spirin A.S. (1975). Mol. Gen. Genet..

[60] Hitz H., Schafer D., Wittmann-Liebold B. (1975). FEBS Lett..

[61] Hitz H., Schäfer D., Wittmann-Liebold B. (1977). Eur. J. Biochem..

[62] Schnier J., Kitakawa M., Isono K. (1986). Mol. Gen. Genet..

[63] Reeh S., Pedersen S. (1979). Mol. Gen. Genet..

[64] Kang W.K., Icho T., Isono S., Kitakawa M., Isono K. (1989). Mol. Gen. Genet..

[65] Kade B., Dabbs E.R., Wittmann-Liebold B. (1980). FEBS Lett..

[66] Kitakawa M., Blumenthal L., Isono K. (1980). Mol. Gen. Genet..

[67] Koonin E.V., Bork P., Sander C. (1994). Nucl. Acids Res..

